# Comparison of Posttraumatic Stress Disorder Checklist Instruments From *Diagnostic and Statistical Manual of Mental Disorders, Fourth Edition *vs *Fifth Edition* in a Large Cohort of US Military Service Members and Veterans

**DOI:** 10.1001/jamanetworkopen.2021.8072

**Published:** 2021-04-27

**Authors:** Cynthia A. LeardMann, Hope Seib McMaster, Steven Warner, Alejandro P. Esquivel, Ben Porter, Teresa M. Powell, Xin M. Tu, William W. Lee, Rudolph P. Rull, Charles W. Hoge

**Affiliations:** 1 Deployment Health Research Department, Naval Health Research Center, San Diego, California; 2 Leidos, San Diego, California; 3 Social Science Research Center, Mississippi State University, Starkville; 4 Division of Biostatistics and Bioinformatics, Department of Family Medicine and Public Health, University of California, San Diego, La Jolla; 5 Center for Psychiatry and Neuroscience, Walter Reed Army Institute of Research, Silver Spring, Maryland; 6 Office of the Army Surgeon General, Falls Church, Virginia

## Abstract

**Importance:**

The definition of posttraumatic stress disorder (PTSD) changed markedly between the *Diagnostic and Statistical Manual of Mental Disorders, Fourth Edition (DSM-IV)* and *DSM-5*, creating challenges for studies and in medical settings spanning this transition.

**Objective:**

To evaluate the ability to compare and assess PTSD, based on *DSM-IV* and *DSM-5* criteria, using PTSD Checklists (PCLs).

**Design, Setting, and Participants:**

This diagnostic study was conducted with survey data collected in October 2019, from the Millennium Cohort Study, a population-based US military cohort study. The population for the present study was restricted to a subset of initial web responders of the 2019 survey cycle, randomly assigned to 1 of 4 survey groups.

**Exposures:**

Each group received the *DSM-IV* and *DSM-5* PCL (PCL–Civilian [PCL-C] version and PCL for *DSM-5* [PCL-5]). PCL instruments were counterbalanced to control for order effects.

**Main Outcomes and Measures:**

Survey data were used to assess PTSD (using the PCL-C and PCL-5), major depressive disorder (using the Patient Health Questionnaire), generalized anxiety (using the Generalized Anxiety Disorder scale), and problem drinking (using the Patient Health Questionnaire). Demographic and military characteristics included age, sex, race/ethnicity, marital status, education, service branch, pay grade, enrollment panel, and military service status.

**Results:**

Among the 1921 participants (mean [SD] age, 50.1 [12.5] years), 1358 (70.7%) were men, 1638 (85.3%) were non-Hispanic White individuals, 1440 (75.0%) were married, and 1190 (61.9%) had at least a bachelor’s degree; 295 (15.4%) had probable PTSD according to *DSM-IV* criteria with PCL-C compared with 286 (14.9%) using *DSM-5* criteria with PCL-5 (κ = 0.77). There was substantial agreement between PCLs for probable PTSD based on *DSM-IV* criteria (295 [15.4%] with PCL-C; 316 [16.4%] with PCL-5; κ = 0.80) and *DSM-5* criteria (286 [14.9%] with PCL-5; 258 [13.4%] with PCL-C; κ = 0.77). Estimated PTSD sum scores showed excellent agreement with observed scores. Using an established crosswalk, PCL-5 sum scores estimated with the PCL-C were similar to observed PCL-5 scores. Of the 17 corresponding items between the 2 instruments, 16 had substantial agreement. Appending 2 additional PCL-C items to the PCL-5 did not significantly alter estimates. The PCL-C and PCL-5 had nearly identical associations with comorbid conditions.

**Conclusions and Relevance:**

The findings of this diagnostic study suggest that PTSD can be successfully assessed and compared over time with either PCL instrument in veteran and military populations.

## Key Points

**Question:**

How well can posttraumatic stress disorder (PTSD) be assessed and compared spanning the transition between the *Diagnostic and Statistical Manual of Mental Disorders, Fourth Edition (DSM-IV)* and *DSM-5*, using different PTSD Checklist (PCL) instruments?

**Findings:**

In this diagnostic study of 1921 individuals, there was substantial to excellent agreement when individual items, probable PTSD, and sum scores were compared between PCL-Civilian (PCL-C) version and PCL for *DSM-5* (PCL-5); the 2 instruments had nearly identical associations with comorbid conditions.

**Meaning:**

These results provide support for the transition from the PCL-C to the PCL-5 without losing the ability to monitor trends and associated comorbidities.

## Teaser

This diagnostic study examines the utility and accuracy of a checklist for posttraumatic stress disorder (PTSD) based on diagnostic criteria from *DSM-IV* vs diagnostic criteria from *DSM-5*.

## Introduction

The American Psychiatric Association’s *Diagnostic and Statistical Manual of Mental Disorders, Fifth Edition* (*DSM-5*), released in 2013, provided updated criteria for mental disorders.^1^ Compared with the previous version, *DSM-IV*-*TR*,^2^ the *DSM-5* made a number of notable revisions to the posttraumatic stress disorder (PTSD) diagnostic criteria, including adding new symptoms, modifying existing symptoms, and dividing the avoidance cluster into avoidance and negative alterations in cognitions and mood. These changes created challenges for longitudinal research and in medical settings, where it is important to maintain consistency in assessment of PTSD and retain the ability to monitor changes over time.

Although the Clinician-Administered PTSD Scale is considered the criterion standard for diagnosing PTSD,^3^ owing to the time and skill required for administration, researchers and clinicians often rely on validated self-reported measures. Based on *DSM-IV/DSM-IV-TR* criteria, the PTSD Checklist (PCL) was the most commonly used instrument; of the 3 versions, PCL–Specific, PCL–Military, and PCL-Civilian (PCL-C), the PCL-C has been used most frequently. Concurrent with changes in diagnostic criteria, the PCL for *DSM-5* (PCL-5) was created to align with the new PTSD criteria.^4^ This 20-item instrument has demonstrated excellent psychometric properties.^5,6,7^ However, there are notable differences between instruments. The PCL-5 includes 3 additional items assessing the novel *DSM-5* symptoms (persistent trauma-related negative emotions, persistent blame, and reckless or self-destructive behavior).^4,8^ Of the remaining 17 items, the wording of 13 was modified to align with the *DSM-5* changes that attempted to clarify symptom expression (11 slightly to moderately modified and 2 heavily modified).

Owing to these differences, the National Center for PTSD stated that “PCL-5 scores are not compatible with PCL for *DSM-IV* scores and cannot be used interchangeably,”^4^ and it has been recommended to administer 2 separate instruments when there is need to assess both sets of *DSM* PTSD criteria. However, this approach is not practical and doubles the burden on individuals. The redundancy of items could lead to frustration, problems with repeated testing, and order effects.^9^ Thus, it is imperative to determine how to assess PTSD based on *DSM-IV* and *DSM-5* criteria with either instrument.

Previous comparisons of the *DSM-IV* and *DSM-5* PCL instruments among soldiers, veterans, and trauma survivors have shown substantial overlap between the measures.^9,10,11,12^ The estimated prevalence of PTSD was found to be similar, and the 2 definitions had nearly identical associations with other psychiatric disorders and functional impairment. Direct comparison of individual items indicated substantial agreement (κ > 0.60) for 16 of the 17 corresponding items among soldiers,^9^ although agreement was lower among trauma survivors (κ = 0.42-0.58).^12^ Among the trauma survivors, a 20-item blended version of the PCL demonstrated excellent internal consistency and correlation with *DSM-IV* and *DSM-5* PCL instruments.^12^ Although these studies provide promising evidence that the 2 instruments have similar psychometric properties, questions remain about the ability to make meaningful clinical comparisons across time for military, veteran, and civilian populations. In 1 study,^9^ for example, approximately 30% of soldiers who met criteria using 1 measure did not meet criteria using the other measure, despite highly comparable psychometric properties. Furthermore, both versions of the instrument are still used in research and clinical settings. Within the US Department of Defense, the PCL-5 has become the standard for assessing symptoms in mental health clinics; however, the *DSM-IV* PCL versions are still routinely used for screening purposes for deployment and annual periodic health assessments.^13^ Although PTSD screening is becoming routine in the US Department of Defense, Veterans Health Administration, and other health care settings, the marked changes in definition and screening instruments over time pose challenges to the evidence foundation for these public health practices. Most evidence-based treatments for PTSD have been validated with older definitions. Therefore, it is crucial that researchers, clinicians, and medical professionals have clear guidance on transitioning between definitions and interpreting data over time that may have involved both instruments.

Using data from a subset of participants from the Millennium Cohort Study who were administered both the PCL-C and PCL-5, the study addressed 5 objectives: (1) determine agreement between individual items on each instrument, (2) assess agreement of probable PTSD and PTSD sum scores using different instruments, (3) assess whether appending the 2 heavily modified PCL-C items to the PCL-5 significantly changed the percentage of participants with probable PTSD, (4) compare the association of PTSD with comorbidities by using both instruments, and (5) determine the agreement between estimated and observed PCL-5 sum scores by using an established crosswalk.^11^ This study was designed to test the hypothesis that there is substantial agreement between the PCL-C and PCL-5 and that *DSM-IV* and *DSM-5* PTSD can be assessed with either instrument. Results from this study will be invaluable in making comparisons across studies and longitudinal efforts and of particular relevance for assessing PTSD among military and veteran populations.

## Methods

### Population and Data Sources

Launched in 2001, the Millennium Cohort Study was designed to examine the long-term health effects associated with military service.^14,15^ Between July 1, 2001, and April 4, 2013, 4 separate cohorts of service members, referred to as panels, were randomly selected from military rosters to participate in the study. Participants were selected from the Army, Navy, Air Force, Marine Corps, and Coast Guard (including active duty, National Guard, and reserve personnel). Enrolled participants (approximately 200 000) were requested to complete a web-based or paper survey at baseline and then every 3 to 5 years during and after leaving military service. A detailed description of the study methods has been published elsewhere.^14,15^ Participants provided voluntary, informed consent. The study was approved by the Naval Health Research Center institutional review board. This study followed the Reporting of studies Conducted Using Observational Routinely Collected Data (RECORD) reporting guideline.

The 2019-2021 follow-up survey transitioned from the PCL-C to the PCL-5. To examine the agreement between these measures, a sample of initial web responders was randomly assigned to 1 of 4 survey groups (labeled A1, A2, B1, and B2) that received both the PCL-C and PCL-5 within the overall questionnaire (separated by 21 web pages or more, depending on skip patterns). Two survey groups (A1 and A2) received the PCL-C and PCL-5, whereas the other 2 groups (B1 and B2) received the PCL-C and the PCL-5 with 2 additional PCL-C items, referred to as the expanded PCL-5. The 2 additional items (“Feeling as if your future will somehow be cut short” and “Feeling emotionally numb, or being unable to have loving feelings for those close to you”) were the items most significantly modified. Survey groups A1 and B1 received the PCL-5 first and the PCL-C as the second instrument, whereas this order was reversed for groups A2 and B2. This counterbalancing of the 2 PCL instruments was designed to control for order effects; the surveys were otherwise identical (Table 1).

**Table 1.** Study Design With Prevalence of Probable PTSD for Each Instrument

Survey group^a^	Sample, No.	First instrument		Second instrument	
		Instrument	Probable PTSD, No. (%)	Instrument	Probable PTSD, No. (%)
A1	475	PCL-5^b^	76 (16.0)	PCL-C^c^	69 (14.5)
A2	479	PCL-C^c^	76 (15.9)	PCL-5^b^	68 (14.2)
B1	490	PCL-5 with 2 PCL-C items^b^	68 (13.9)	PCL-C^c^	61 (12.5)
B2	477	PCL-C^c^	89 (18.7)	PCL-5 with 2 PCL-C items^b^	74 (15.5)

Abbreviations: PCL-C, PTSD Checklist–Civilian; PCL-5, PTSD Checklist for *DSM-5*; PTSD, posttraumatic stress disorder.

^a^ Instructions for both PCL instruments were the same: “Below is a list of problems that people sometimes have in response to a very stressful experience. Please indicate how much you have been bothered by each problem in the past month.”

^b^ Assessed according to endorsing moderate or higher for at least 1 intrusion symptom, 1 avoidance symptom, 2 negative alterations in cognitions and mood, and 2 hyperarousal symptoms, using the 20 PCL-5 items.

^c^ Assessed according to endorsing moderate or higher for at least 1 intrusive reexperiencing symptom, 3 avoidance symptoms, and 2 hyperarousal symptoms, using the 17 PCL-C items.

Once survey completions for each group passed 500, random assignment was halted, which occurred within a week after the survey cycle opened. Participants who completed their questionnaire by October 30, 2019, (when data for this project were accessed) were included in the study (n = 2060). After exclusion of participants with missing PCL-C or PCL-5 items (n = 139), the study population consisted of 1921 individuals (Table 1).

### Measures

Instructions for the 2 PCL measures were standardized to ensure comparability. Likewise, response options were identical for both instruments, ranging from not at all to extremely, although scoring was different (PCL-C items scored 1 to 5, range 17-85; PCL-5 items scored 0 to 4, range 0-80). According to *DSM-IV* criteria, participants screened positive for probable PTSD if they reported moderate or higher on at least 1 intrusive reexperiencing symptom, 3 avoidance symptoms, and 2 hyperarousal symptoms.^2^ In accordance with *DSM-5* criteria, participants screened positive for probable PTSD if they reported moderate or higher on at least 1 intrusion symptom (previously reexperiencing), 1 avoidance symptom, 2 negative alterations in cognitions or mood, and 2 hyperarousal symptoms.^1^

Major depressive disorder was measured with the 8-item module from the Patient Health Questionnaire,^16^ and generalized anxiety with the 2-item Generalized Anxiety Disorder scale.^17^ The alcohol scale of the Patient Health Questionnaire was used to assess problem drinking (at least 1 of 5 items endorsed).^16^ Demographic and military characteristics (ie, age, sex, race/ethnicity, marital status, education, service branch, pay grade, enrollment panel, and military service status) were assessed via survey and electronic personnel files managed by the Defense Manpower Data Center.

### Statistical Analysis

Descriptive analyses, including frequencies and χ^2^ tests, were used to compare characteristics between survey groups. Order effects, based on percentage of participants with probable PTSD, were assessed with generalized linear models with inference based on generalized estimating equations to improve inference validity,^18^ adjusting for instrument type (PCL-C or PCL-5) and an interaction between order and instrument type.

For determining agreement between individual items on each instrument and assessing agreement of probable PTSD with *DSM-IV* and *DSM-5* criteria on different instruments, Cohen simple κ (for binary data) and weighted κ using Cicchetti-Allison weights (for categorical data) were calculated. Intraclass correlation coefficients (ICCs) were calculated to examine the agreement of PTSD sum scores.^19^ Because the PCL-C is missing 3 items present on the PCL-5, imputation was used to calculate the PCL-5 sum score using PCL-C items. The effectiveness of mean imputation and multiple imputation to recover this missing information was compared. For assessing whether appending the 2 heavily modified PCL-C items to the PCL-5 significantly changed the percentage or sum score of PTSD, χ^2^ and *t* tests were used to compare probable PTSD and PTSD sum scores, respectively, using the 20 PCL-5 items based on *DSM-5* criteria between survey groups A (PCL-5) and B (expanded PCL-5). For comparing the association of PTSD with comorbidities by using both instruments, the prevalence of comorbidities was calculated, and regression coefficients of associations between comorbidities with *DSM-IV* and *DSM-5* criteria were compared. For determining the agreement between estimated and observed PCL-5 sum scores using an established crosswalk, ICCs were calculated.^11^ All analyses were performed with SAS version 9.4 (SAS Institute). Statistical significance was set at *P* < .05, and all tests were 2-tailed.

## Results

The study population of 1921 individuals (mean [SD] age, 50.1 [12.5] years) was predominantly men (1358 [70.7%]), non-Hispanic White individuals (1638 [85.3%]), married (1440 [75.0%]), with at least a bachelor’s degree (1190 [61.9%]), Army service member (843 [43.9%]), enlisted rank (1242 [64.7%]), separated from the military (1490 [77.6%]), and from the first enrollment panel (1210 [63.0%]). Each of the 4 survey groups consisted of approximately one-quarter of the sample, and no differences in demographic or military characteristics across the groups were detected, except for panel (Table 2). For all survey groups, prevalence of probable PTSD was significantly higher with the first PCL instrument than the second (Table 1) irrespective of version (b = 0.29; 95% CI, 0.04 to 0.54; *P* = .02). However, no significant difference in PTSD prevalence was observed by instrument type (b = 0.11; 95% CI, −0.14 to 0.37; *P* = .39) or from the interaction of order and instrument type (b = −0.29; 95% CI, −0.76 to 0.18; *P* = .23).

**Table 2.** Demographic and Military Characteristics by Survey Group

Characteristic	No. (%)^a^							*P* value
	Full sample, No. (%) (N = 1921)	A1 survey group, PCL-5/PCL-C (n = 475)^b^		A2 survey group, PCL-C/PCL-5 (n = 479)^b^		B1 survey group, PCL-5 with 2 PCL-C items/PCL-C (n = 490)^b^	B2 survey group, PCL-C/PCL-5 with 2 PCL-C items (n = 477)^b^	
**Demographic characteristics**								
Age, mean (SD), y	50.1 (12.5)		50.3 (12.0)		50.1 (12.2)	50.8 (13.1)	49.4 (12.5)	.40
Sex								.55
Men	1358 (70.7)		328 (24.2)		343 (25.3)	356 (26.2)	331 (24.4)	
Women	563 (29.3)		147 (26.1)		136 (24.2)	134 (23.8)	146 (25.9)	
Race/ethnicity								
Asian/Pacific Islander	40 (2.1)		6 (15.0)		9 (22.5)	13 (32.5)	12 (30.0)	.41
Black, non-Hispanic	118 (6.1)		25 (21.2)		24 (20.3)	34 (28.8)	35 (29.7)	
White, non-Hispanic	1638 (85.3)		419 (25.6)		409 (25.0)	406 (24.8)	404 (24.7)	
Hispanic	92 (4.8)		18 (19.6)		26 (28.3)	30 (32.6)	18 (19.6)	
Other^c^	33 (1.7)		7 (21.2)		11 (33.3)	7 (21.2)	8 (24.2)	
Marital status								
Not married	481 (25.0)		130 (27.0)		118 (24.5)	114 (23.7)	119 (24.7)	.52
Married	1440 (75.0)		345 (24.0)		361 (25.1)	376 (26.1)	358 (24.9)	
Educational attainment								
≤High school degree	90 (4.7)		20 (22.2)		19 (21.1)	26 (28.9)	25 (27.8)	.52
Some college	403 (21.0)		100 (24.8)		106 (26.3)	106 (26.3)	91 (22.6)	
Associate’s degree	238 (12.4)		48 (20.2)		71 (29.8)	58 (24.4)	61 (25.6)	
Bachelor’s degree	549 (28.6)		137 (25.0)		130 (23.7)	132 (24.0)	150 (27.3)	
≥Master’s degree	641 (33.4)		170 (26.5)		153 (23.9)	168 (26.2)	150 (23.4)	
**Military service**								
Service branch								
Army	843 (43.9)		214 (25.4)		214 (25.4)	209 (24.8)	206 (24.4)	.69
Navy	345 (18.0)		89 (25.8)		95 (27.5)	79 (22.9)	82 (23.8)	
Marine Corps	107 (5.6)		24 (22.4)		30 (28.0)	31 (29.0)	22 (20.6)	
Air Force	590 (30.7)		137 (23.2)		132 (22.4)	161 (27.3)	160 (27.1)	
Coast Guard	34 (1.8)		10 (29.4)		8 (23.5)	9 (26.5)	7 (20.6)	
Pay grade								
Junior enlisted and NCOs	1242 (64.7)		298 (24.0)		310 (25.0)	312 (25.1)	322 (25.9)	.45
Officer	679 (35.3)		177 (26.1)		169 (24.9)	178 (26.2)	155 (22.8)	
Service status								
Regular active	199 (10.4)		38 (19.1)		48 (24.1)	61 (30.7)	52 (26.1)	.35
Reserve or National Guard	228 (11.9)		59 (25.9)		51 (22.4)	62 (27.2)	56 (24.6)	
Veteran	1490 (77.6)		376 (25.2)		380 (25.5)	365 (24.5)	369 (24.8)	
**Millennium Cohort Study characteristics**								
Panel								
1	1210 (63.0)		308 (25.5)		310 (25.6)	303 (25.0)	289 (23.9)	.02
2	225 (11.7)		55 (24.4)		45 (20.0)	73 (32.4)	52 (23.1)	
3	219 (11.4)		63 (28.8)		55 (25.1)	42 (19.2)	59 (26.9)	
4	267 (13.9)		49 (18.4)		69 (25.8)	72 (27.0)	77 (28.8)	

Abbreviations: NCO, noncommissioned officer; PCL-C, PTSD Checklist–Civilian; PCL-5, PTSD Checklist for *DSM-5*.

^a^ Percentages for full sample are column percentages, while those for the survey groups are percentages of the row.

^b^ Survey group was based on which PCL instruments were received and order of the instruments. Survey groups were as follows: A1, PCL-5 followed by PCL-C; A2, PCL-C followed by PCL-5; B1, PCL-5 plus 2 PCL-C items followed by PCL-C; and B2, PCL-C followed by PCL-5 plus 2 PCL-C items.

^c^ Other includes individuals who identify as American Indian or mixed race/ethnicity.

When the individual items from the PCL-C vs PCL-5 were compared, 1 had moderate agreement (“Feeling as if your future will somehow be cut short”; weighted κ = 0.52), whereas the other 16 items had substantial agreement (weighted κ = 0.60-0.80) (Table 3). When endorsement of the items was based on a rating of moderate or higher, results indicated slightly better agreement than the results from the weighted κ (κ = 0.61-0.81; data not shown). For participants in group B (completed expanded PCL-5), there was also strong agreement (weighted κ = 0.75 and 0.77) between the 2 PCL-C items appended to the PCL-5 scale and those same questions within the PCL-C.

**Table 3.** Direct Comparison of Individual Items From the PCL-5 and PCL-C

PCL-5 item	PCL-C item	Weighted κ
**Criterion B (intrusion)**		
Repeated, disturbing, and unwanted memories of the stressful experience	Repeated, disturbing memories or stressful experiences from the past	0.72
Repeated, disturbing dreams of the stressful experience	Repeated, disturbing dreams of stressful experiences from the past	0.78
Suddenly feeling or acting as if the stressful experience were actually happening again (as if you were actually back there reliving it)	Suddenly acting or feeling as if stressful experiences were happening again	0.71
Feeling very upset when something reminded you of the stressful experience	Feeling very upset when something happened that reminds you of stressful experiences from the past	0.74
Having strong physical reactions when something reminded you of the stressful experience (for example, heart pounding, trouble breathing, sweating)	Physical reactions when something reminds you of stressful experiences from the past	0.72
**Criterion C (avoidance)**		
Avoiding memories, thoughts, or feelings related to the stressful experience	Avoid thinking about your stressful experiences from the past or avoid having feelings about them	0.70
Avoiding external reminders of the stressful experience (for example, people, places, conversations, activities, objects, or situations)	Avoid activities or situations because they remind you of stressful experiences from the past	0.72
**Criterion D (negative alterations in cognitions and mood)**		
Trouble remembering important parts of the stressful experience	Trouble remembering important parts of stressful experiences from the past	0.72
Having strong negative beliefs about yourself, other people, or the world (for example, having thoughts such as I am bad, there is something seriously wrong with me, no one can be trusted, the world is completely dangerous)	Feeling as if your future will somehow be cut short	0.52
Blaming yourself or someone else for the stressful experience or what happened after it	NA	NA
Having strong negative feelings such as fear, horror, anger, guilt, or shame	NA	NA
Loss of interest in activities that you used to enjoy	Loss of interest in activities that you used to enjoy	0.75
Feeling distant or cut off from other people	Feeling distant or cut off from other people	0.74
Trouble experiencing positive feelings (for example, being unable to feel happiness or have loving feelings for people close to you)	Feeling emotionally numb, or being unable to have loving feelings for those close to you	0.68
**Criterion E (hyperarousal)**		
Irritable behavior, angry outbursts, or acting aggressively	Feeling irritable or having angry outbursts	0.65
Taking too many risks or doing things that could cause you harm	NA	NA
Being “superalert” or watchful or on guard	Feeling “superalert” or watchful or on guard	0.77
Feeling jumpy or easily startled	Feeling jumpy or easily startled	0.80
Having difficulty concentrating	Difficulty concentrating	0.71
Trouble falling or staying asleep	Trouble falling asleep or staying asleep	0.73

Abbreviations: NA, not applicable; PCL-C, PTSD Checklist–Civilian; PCL-5, PTSD Checklist for *DSM-5*.

Of the 1921 participants, 295 (15.4%) had probable PTSD according to *DSM-IV* criteria with the 17 PCL-C items and 286 (14.9%) according to *DSM-5* criteria with the 20 PCL-5 items (κ = 0.77) (Table 4), indicating substantial agreement. Although the prevalence was similar, 60 of the 295 participants (20.3%) who met *DSM-IV* PTSD criteria with PCL-C did not meet *DSM-5* criteria with the PCL-5. Similarly, of 286 participants who met *DSM-5* criteria with PCL-5, 51 (17.8%) did not meet *DSM-IV* criteria with PCL-C. When specific cutoffs were examined (data not shown), the recommended PCL-5 cutoffs^7^ of 31 to 33 had strongest agreement with PCL-C cutoffs of 42 and 43 (κ = 0.82-0.85). A PCL-5 cutoff of 18 and 39 corresponded most closely to the PCL-C cutoffs of 35 and 50 (κ = 0.86 and 0.81, respectively).

**Table 4.** Prevalence of Probable Posttraumatic Stress Disorder by Scoring Criteria and PCL Instrument (N = 1921)

Criteria used	No. (%)		Cohen κ
	PCL-C	PCL-5	
*DSM-IV* vs *DSM-5*	295 (15.4)^a^	286 (14.9)^b^	0.77
*DSM-IV*	295 (15.4)^a^	316 (16.4)^a^^,^^c^	0.80
*DSM-5*	258 (13.4)^b^^,^^d^	286 (14.9)^b^	0.77

Abbreviations: *DSM, Diagnostic and Statistical Manual of Mental Disorders*; PCL-C, PTSD Checklist–Civilian; PCL-5, PTSD Checklist for *DSM-5*.

^a^ Assessed according to endorsing moderate or higher for at least 1 instrusive reexperiencing symptom, 3 avoidance symptoms, and 2 hyperarousal symptoms (*DSM-IV* criteria).

^b^ Assessed according to endorsing moderate or higher for at least 1 intrusion symptom, 1 avoidance symptom, 2 negative alterations in cognitions and mood, and 2 hyperarousal symptoms (*DSM-5* criteria).

^c^ *DSM-IV* criteria assessed with the 17 PCL-5 items that correspond to the PCL-C items.

^d^ *DSM-5* criteria assessed with the 17 PCL-C items that correspond to the PCL-5 items.

Using *DSM-IV* criteria to assess probable PTSD, there was substantial agreement (PCL-C, 295 [15.4%]; PCL-5, 316 [16.4%]; κ = 0.80) (Table 4). Using data from survey group B, the expanded PCL-5 had almost perfect agreement with the PCL-C according to *DSM-IV* criteria (PCL-C, 150 [15.5%]; expanded PCL-5, 153 [15.8%]; κ = 0.83). There was also substantial agreement with *DSM-5* criteria to assess probable PTSD by using the 20 PCL-5 items compared with 17 PCL-C items, with the other 3 items treated as missing (PCL-5, 286 [14.9%]; PCL-C, 258 [13.4%]; κ = 0.77) (Table 4). When PCL-C sum scores were estimated with 17 items from the PCL-5, there was excellent reliability between the observed sum scores (mean [SD], 29.1 [15.1]) and estimated sum scores (mean [SD], 29.0 [15.1]; ICC, 0.95). The estimated PCL-5 sum scores, based on the 17 PCL-C items, using multiple imputation (mean [SD], 13.6 [17.3]; ICC, 0.95) and mean imputation (mean [SD], 14.3 [17.7]; ICC, 0.95) were similar to the observed PCL-5 sum score (mean [SD], 13.5 [17.4]).

Using the 20 PCL-5 items, the percentage of participants with probable *DSM-5* PTSD was not significantly different between group A (PCL-5, 144 [15.1%]) and group B (expanded PCL-5, 142 [14.7%]; *P* = .80). There was no significant difference between the sum scores (group A: mean [SD], 13.5 [17.7]; group B, mean [SD], 13.4 [17.2]; *P* = .94).

In examining the association of comorbidities with PTSD, the proportions with comorbidities were highly comparable, with no statistical differences between the *DSM-IV* and *DSM-5* instruments and criteria (Table 5). When the crosswalk by Moshier et al^11^ was implemented, there was excellent agreement (ICC, 0.95) between estimated (mean [SD], 12.9 [17.2]) and observed (mean [SD], 13.5 [17.4]) PCL-5 sum scores. When stratified, agreement remained excellent for both veterans (observed mean [SD], 14.1 [17.8]; estimated mean [SD], 13.5 [17.6]; ICC, 0.95) and service members (observed mean [SD], 11.3 [15.9]; estimated mean [SD], 10.9 [15.5]; ICC, 0.93).

**Table 5.** Comorbid Conditions Using the PTSD Checklist for *DSM-5* (PCL-5) and PCL-Civilian (PCL-C) Version

Condition^a^	No. (%)		*P* value^d^
	PCL-C^b^	PCL-5^c^	
Major depression^e^			
Major depression with PTSD	159 (54.3)	143 (50.4)	.35
Major depression with no PTSD	26 (1.6)	42 (2.6)	
Problem drinking^f^			
Problem drinking with PTSD	60 (26.0)	57 (25.3)	.88
Problem drinking with no PTSD	105 (7.7)	108 (7.9)	
GAD^g^			
GAD with PTSD	177 (63.4)	174 (64.0)	.90
GAD with no PTSD	79 (5.1)	82 (5.3)	

Abbreviations: GAD, generalized anxiety disorder; DSM, *Diagnostic and Statistical Manual of Mental Disorders*; PCL-C, PTSD Checklist–Civilian; PCL-5, PTSD Checklist for *DSM-5*; PTSD, posttraumatic stress disorder.

^a^ Based on missing data, study sample varied slightly for each comorbid condition (major depression, n = 1919; problem drinking, n = 1598; GAD, n = 1821).

^b^ Assessed according to endorsing moderate or higher for at least 1 intrusive reexperiencing symptom, 3 avoidance symptoms, and 2 hyperarousal symptoms (*DSM-IV* criteria).

^c^ Assessed according to endorsing moderate or higher for at least 1 intrusion symptom, 1 avoidance symptom, 2 negative alterations in cognitions and mood, and 2 hyperarousal symptoms (*DSM-5* criteria).

^d^ Determined from directly comparing regression coefficients between PTSD criteria and outcomes.

^e^ Classified individuals who endorsed 5 or more of the 8 items as more than half the days or nearly every day, including the endorsement of anhedonia or depressed mood.

^f^ Assessed according to a positive endorsement of any of the 5 Patient Health Questionnaire alcohol items (eg, drank while working or taking care of responsibilities, drove a car after drinking too much).

^g^ Assessed with the standard scoring algorithm (scoring ≥3 points) of the GAD scale.

## Discussion

This study of nearly 2000 US service members and veterans extends previous research comparing PTSD definitions using different PCL instruments. The results indicated that the PCL-C and PCL-5 yielded remarkably similar outcomes with both *DSM-IV* and *DSM-5* criteria. The 17-item PCL-C was able to effectively estimate PCL-5 sum scores and PTSD prevalence (based on *DSM-5* criteria); similarly, PCL-C sum scores and prevalence (based on *DSM-IV* criteria) were effectively estimated with the PCL-5. The addition of the 2 PCL-C items to the PCL-5 did not significantly alter estimates and thus may represent an unnecessary, albeit benign, addition. Last, the PCL-C and PCL-5 showed nearly identical associations with comorbid conditions. These findings were further strengthened by counterbalancing the order of PCL instruments. Taken together, these findings suggest that research and medical settings will successfully be able to assess PTSD over time among service members and veterans with these different instruments.

In settings in which it is important to maintain consistency in assessment of PTSD, it is essential to be able to apply data from the *DSM-IV* PCL to estimate *DSM-5* PTSD. This study demonstrated 2 applicable techniques. First, responses from the PCL-C were recoded to align with 17 corresponding PCL-5 items, and the 3 missing items were imputed. The estimated sum scores had excellent agreement with the observed PCL-5 sum scores. Although mean imputation is slightly less accurate, using it rather than multiple imputation would simplify analyses without creating substantial misclassification, although it may underestimate standard errors. Second, a previously established crosswalk^11^ was used to estimate PCL-5 scores based on PCL-C sum scores. Estimated PCL-5 scores aligned closely with the observed PCL-5 scores, performing comparably well in this study population. Thus, this crosswalk appears to be generalizable to both service members and veterans, suggesting either of these approaches may be used for research studies and surveillance efforts.

Although most settings will likely rely on *DSM-5* criteria, there may be certain instances in which *DSM-IV* PTSD criteria still need to be estimated, for example, for comparison or replication purposes. The current study found that the estimated prevalence of *DSM-IV* PTSD and sum scores were similar when the PCL-5 was used compared with PCL-C instruments. Because the expanded PCL-5 did not offer a meaningful advantage over the original PCL-5, the 20-item PCL-5 can be used to estimate PTSD with *DSM-5* or *DSM-IV* criteria.

Findings from this cohort add to prior comparisons.^9,11,12^ A study of soldiers found the PCL-5 to be highly comparable to the 17-item PCL–Specific.^9^ Both that study^9^ and this one found substantial agreement for 16 of 17 corresponding PCL items and no significant differences in prevalence of comorbidities of depression, generalized anxiety, or alcohol problems with PTSD measured with *DSM-IV* and *DSM-5* criteria.^9^ The discordance between instruments was also similar, although slightly lower among our study population. Taken together, these results suggest that the 2 scales are comparable for epidemiologic and clinical purposes, and replicate prior findings suggesting that the 2 definitions have comparable clinical utility with no clear advantage afforded by the changes that were made.^9,20^

### Limitations

A key limitation of this analysis was the absence of a clinical criterion-standard diagnosis of PTSD, which was not feasible in such a large population-based study. However, the PCL is a widely used instrument in clinical settings and research, and the true diagnostic status of participants would not change the agreement or comparison between PCL instruments. The sample was predominantly non-Hispanic White individuals and men, which may limit generalizability. The sample included both veterans and service members from all branches and components, and investigations of the Millennium Cohort Study have not demonstrated systematic sampling bias.^21,22^

## Conclusions

The current findings add to an increasing body of literature suggesting that the PCL-5 can be used to estimate *DSM-IV* PTSD and the PCL-C can be used to estimate *DSM-5* PTSD. These results provide strong support for the transition from the 17-item PCL to the 20-item PCL-5 without losing the ability to monitor trends and associated comorbidities over time.

## Notes

Supplement. **Nonauthor Collaborators.** Millennium Cohort Study Team
