# Supplementary material for: Comparison of Posttraumatic Stress Disorder Checklist Instruments From Diagnostic and Statistical Manual of Mental Disorders, Fourth Edition vs Fifth Edition in a Large Cohort of US Military Service Members and Veterans
Source: JAMA Netw Open. 2021 Apr 27;4(4):e218072. doi: 10.1001/jamanetworkopen.2021.8072 (PMC8080232; doi:10.1001/jamanetworkopen.2021.8072)
Supplement: Supplement. — Nonauthor Collaborators. Millennium Cohort Study Team [file jamanetwopen-e218072-s001.pdf]

Supplemental Online Content: Nonauthor Collaborators

*Indicates required information. Only first name, last name, and suffix will appear in PubMed.

***Group Name(s): Millennium Cohort Study team**							
***First Name and Middle Initial(s)**	***Last Name**	***Suffix (eg, Jr, III)**	Academic Degrees	Institution	Location (city, state/province, country)	Role or Contribution, eg, chair, principal investigator	Group (if more than 1 Group listed in the byline) and/or Subgroup (eg, Steering Committee)
Satbir K.	Boparai		MBA	NHRC	San Diego, CA		
Felicia R.	Carey		PhD	NHRC	San Diego, CA		
Sheila F.	Castañeda		PhD	NHRC	San Diego, CA		
Toni Rose T.	Geronimo-Hara		MPH	NHRC	San Diego, CA		
Isabel G.	Jacobson		MPH	NHRC	San Diego, CA		
Claire A.	Kolaja		MPH	NHRC	San Diego, CA		
Rayna K.	Matsuno		PhD	NHRC	San Diego, CA		
Deanne C.	Millard		BA	NHRC	San Diego, CA		
Anna C.	Rivera		MPH	NHRC	San Diego, CA		
Beverly D.	Sheppard		BS	NHRC	San Diego, CA		
Daniel W.	Trone		PhD	NHRC	San Diego, CA		
Jennifer L.	Walstrom			NHRC	San Diego, CA		
